# Interstitial Ectopic Pregnancy Associated With Painless and Severe Vaginal Bleeding: A Rare, Atypical Clinical Presentation

**DOI:** 10.7759/cureus.53225

**Published:** 2024-01-30

**Authors:** Anna Thanasa, Efthymia Thanasa, Vasiliki Grapsidi, Ioannis-Rafail Antoniou, Ektoras-Evangelos Gerokostas, Evangelos Kamaretsos, Athanasios Chasiotis, Ioannis Thanasas

**Affiliations:** 1 Medicine, Department of Health Sciences, School of Medicine, Aristotle University of Thessaloniki, Thessaloniki, GRC; 2 Obstetrics and Gynecology, General Hospital in Trikala, Trikala, GRC; 3 Obstetrics and Gynecology, General Hospital of Trikala, Trikala, GRC; 4 Obstetrics and Gynecology, Limassol General Hospital, Limassol, CYP

**Keywords:** interstitial ectopic pregnancy, transvaginal ultrasound, doppler ultrasound, magnetic resonance imaging, chorionic gonadotropin hormone, medical treatment, surgical treatment, case report

## Abstract

Interstitial ectopic pregnancy is rare (2%-4% of ectopic pregnancies). The atypical clinical presentation of interstitial ectopic pregnancy associated with massive vaginal bleeding is extremely rare and makes early preoperative diagnosis even more difficult. The presentation of our case concerns the early diagnosis and surgical treatment of a patient with an interstitial ectopic pregnancy without rupture, which presented atypically with painless, severe vaginal bleeding. A 27-year-old fourth-term pregnant woman presented with massive painless vaginal bleeding. Secondary amenorrhea was calculated at eight weeks and four days. Transvaginal ultrasound and transvaginal Doppler ultrasound combined with the quantification of beta-chorionic gonadotropin hormone raised the suspicion of interstitial ectopic pregnancy. Intraoperatively, the presence of a large swelling of the right horn of the uterus was established, and a wedge resection was performed with the removal of the corresponding fallopian tube. Three weeks after surgery, the serum beta-chorionic gonadotropin hormone value was zero. In this paper, the rarity of interstitial ectopic pregnancy, the difficulties related to early and correct preoperative diagnosis, and the selection of the appropriate available therapeutic procedures are emphasized, the correct application of which can significantly contribute to reducing the morbidity and mortality of these patients.

## Introduction

Ectopic pregnancy is defined as a pregnancy in which the implantation of the fertilized egg occurs outside the normal uterine cavity [1]. Ectopic pregnancy has become more prevalent, particularly with the widespread adoption of assisted reproductive techniques. The incidence of ectopic pregnancies has seen a notable rise, escalating from 2%, which is after natural conception, to 8.6% in pregnancies resulting from in vitro fertilization after embryo transfer [2]. Also, despite the widespread availability of modern diagnostic and therapeutic modalities in recent years, ectopic pregnancy remains the most common cause of maternal mortality during the first trimester of pregnancy. It is estimated to account for 5%-10% of all pregnancy-related deaths [3].

The most common type of ectopic pregnancy is tubal, most often located in the ampulla and rarely in the interstitial part of the fallopian tube (interstitial ectopic pregnancy). Non-tubal locations of the disease (cervical, ovarian, and abdominal pregnancies) are rare and involve about 5% of all ectopic pregnancies [3, 4]. In an interstitial pregnancy, the fertilized egg settles outside the uterine cavity in the epithelium of the interstitial part of the fallopian tube. This segment of the fallopian tube passes through the uterine wall to reach the intrauterine cavity via the proximal tubal [5, 6]. Interstitial ectopic pregnancy is rare, accounting for 2%-4% of all ectopic pregnancies [3]. Interstitial ectopic pregnancy should not be confused with angular pregnancy or cornual pregnancy, as they are not ectopic pregnancies [7]. In angular pregnancy, which can be viable, the implantation of the fertilized egg occurs at the lateral angle of the intrauterine cavity, specifically at the junction where the proximal tubal is located [8]. In cornual pregnancy, the implantation of the fertilized egg takes place in the cavity of a rudimentary uterine horn, which may or may not communicate with the main intrauterine cavity [9].

This case report highlights the rarity of interstitial ectopic pregnancy, particularly when associated with painless, severe vaginal bleeding. Furthermore, the difficulties related to early and accurate preoperative diagnosis are emphasized, which is considered to contribute significantly to the choice of optimal therapeutic management and the reduction of morbidity and mortality in these patients.

## Case presentation

The case report concerns a 27-year-old patient with three vaginal deliveries following natural conception and a medical history of pelvic inflammatory disease who presented to the emergency department of the General Hospital of Trikala, Trikala, Greece, due to severe vaginal bleeding. The patient, without ever having had an intrauterine contraceptive coil in the past, was diagnosed three years ago with pelvic inflammatory disease based on clinical findings, for which she needed to be hospitalized three times. The bleeding was not accompanied by abdominal pain. The patient reported mild discomfort in the lower abdomen for the past 10 days, with a constant intensity of two out of 10 on the numerical pain rating scale. Upon physical examination, the abdomen was soft, non-tender, and painless on palpation. Based on the last normal menstrual period, secondary amenorrhea was estimated at eight weeks and four days. The urine pregnancy test done at the hospital was positive. Laboratory testing was normal (Table 1).

**Table 1:** Preoperative and postoperative laboratory tests of the patient diagnosed with an unruptured interstitial ectopic pregnancy Ht: hematocrit; Hb: hemoglobin; PLT: platelets; WBC: white blood cells; NEUT: neutral; APTT: activated partial thromboplastin time; INR: international normalized ratio; FIB: fibrinogen; U: Urea, Cr: creatinine; β-Hgh: beta-chorionic gonadotropin hormone

Laboratory tests	Preoperative values (day of admission)	Preoperative values (48 hours later)	Values on the second postoperative day after the laparotomy	Normal laboratory values
Ht	35.8%	34.7%	33.2%	37.7 – 49.7%
Hb	12.1 gr/dl	11.6 gr/dl	10.3 gr/dl	11.8 – 17.8 gr/dl
PLT	231x10^3^/ml	325x10^3^/ml	210x10^3^/ml	150 – 350 x10^3^/ml
WBC	7.2x40^3^/ml	8.9x10^3^/ml	10.1x10^3^/ml	4 – 10.8 x10^3^/ml
NEUT	64.3%	69.3%	79.5%	40 – 75%
APTT	31.3 sec		32.5 sec	24.0 – 35.0 sec
INR	0.95		1.01	0.8 – 1.2
FIB	350 mg/dl		233 mg/dl	200 – 400 mg/dl
U	21 mg/dl		25 mg/dl	10 – 50 mg/dl
Cr	0.47 mg/dl		0.51 mg/dl	0.40 – 1.10 mg/dl
β-hGH	1140 mlU/mL	1,140 mlU/mL	251 mlU/mL	0 – 5 mlU/mL

The evaluation of the normal progression of the pregnancy through the quantification of beta-chorionic gonadotropin hormone showed a non-viable fetus.

**Figure 1:**
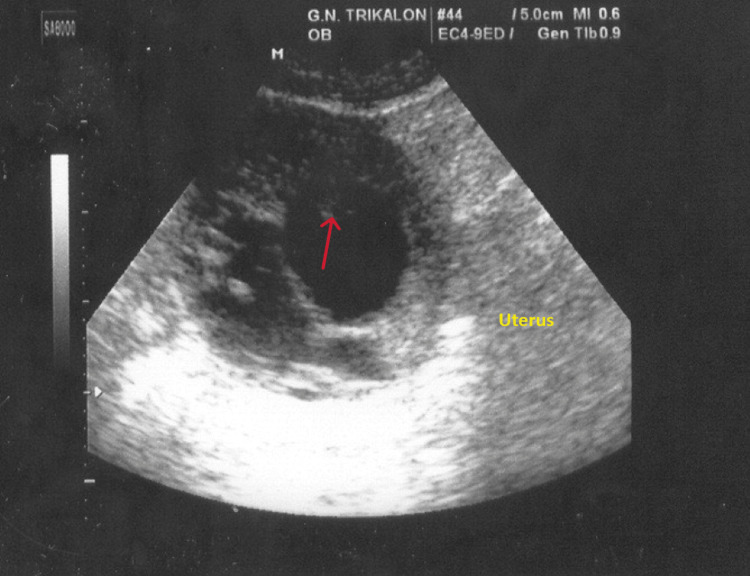
Transvaginal ultrasound imaging showing an interstitial ectopic pregnancy The body of the uterus is visible, as is the presence of a gestational sac with fetal elements without cardiac function (red arrow) in contact with the body of the uterus.

The gynecological clinical examination revealed active uterine bleeding from the external cervical os, which was closed. Mild tenderness on the movement of the cervix was noted during a bimanual pelvic examination. Transvaginal ultrasound revealed the presence of a gestational sac between the uterus and the ovary, which was in contact with the corpus of the uterus (Figure 1).

**Figure 2:**
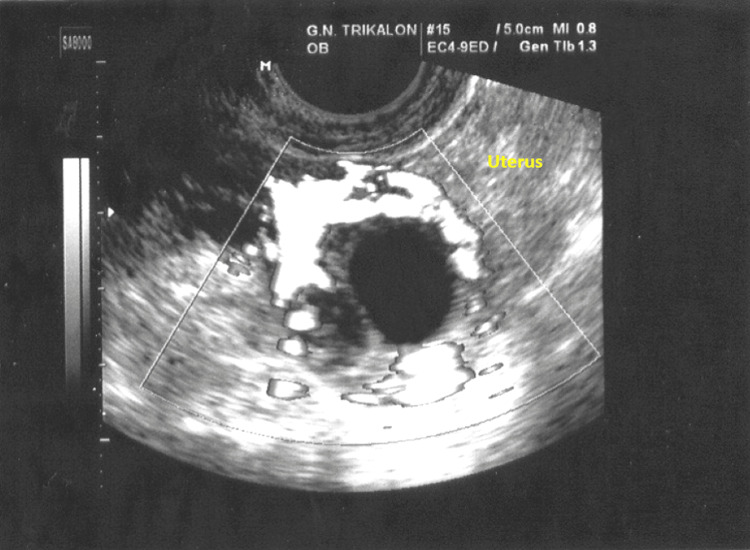
Transvaginal Doppler ultrasound imaging of the interstitial ectopic pregnancy Increased blood flow is an important criterion in the preoperative diagnosis of interstitial ectopic pregnancy.

Also, increased peripheral blood flow was observed in the transvaginal color Doppler ultrasound (Figure 2).

Typical ultrasound imaging, combined with plateau levels of beta-chorionic gonadotropin hormone, raised the suspicion of interstitial ectopic pregnancy. Lacking experience in the conservative management of the patient, involving ultrasound-guided local injection of methotrexate in the gestational sac or systemic administration of methotrexate, the decision was made to surgically investigate the disease through laparotomy. Laparoscopic access was not available at our hospital. During surgery, the presence of an enlargement of the right uterine horn, accompanied by hemorrhagic infiltration and localized ischemic necrosis, without signs of wall rupture, was observed. A wedge resection of the horn was performed simultaneously with the dissection of the ipsilateral fallopian tube.

After a smooth postoperative course, the patient was discharged from our clinic on the fourth postoperative day with a consultation for re-examination at the gynecological outpatient clinic. Three weeks later, the serum beta-chorionic gonadotropic hormone value was negative.

## Discussion

The preoperative diagnosis of interstitial ectopic pregnancy is challenging [10]. Typically, these patients seek medical care relatively late compared to women with tubal pregnancies in other locations. This delay can be attributed to the fact that the presence of the myometrium in the interstitial part of the fallopian tube, which is approximately 0.7 mm in diameter and 1-2 cm in length, provides more space for the growth of the fertilized egg compared to other parts of the fallopian tube [11]. Abdominal pain caused by tissue distension, minor vaginal bleeding resulting from the normal shedding of basal decidua from the intrauterine cavity after the cessation of trophoblastic activity, and secondary amenorrhea constitute the classic triad of symptoms that characterize patients with unruptured interstitial ectopic pregnancy [9]. In cases of rupture of the interstitial part of the fallopian tube and the occurrence of intra-abdominal hemorrhage, the predominant clinical condition is hemorrhagic shock [12, 13]. In our patient, who had nine weeks of amenorrhea and significant tissue distension with the presence of ischemia and localized necrosis in the right uterine horn (Figure 3), unexpected abdominal pain was of mild intensity. Also remarkable was the occurrence of significant uterine bleeding with blood clots, which cannot be attributed to the normal shedding of basal decidua from the intrauterine cavity.

In contrast to the clinical criteria, the contribution of modern imaging modalities in the diagnosis of interstitial ectopic pregnancy seems to be crucial. The current use of transvaginal ultrasonography, three-dimensional ultrasonography, transvaginal color Doppler ultrasonography, and magnetic resonance imaging in combination with the quantification of beta-chorionic gonadotropin hormone levels has remarkably increased the diagnostic accuracy of the disease. Ultrasound findings, including the presence of a gestational sac separated from the intrauterine cavity and surrounded by a thin layer of myometrium (Figure 1 in our case), along with increased peripheral blood flow (Figure 2 in our case), support the diagnosis of interstitial ectopic pregnancy [14]. Li et al., analyzing the results of their study, showed that a transvaginal ultrasound in the diagnosis of interstitial ectopic pregnancy has a sensitivity of 97.8% and a positive predictive value of 99.4% [15]. Additionally, three-dimensional ultrasonography, when compared to transvaginal ultrasonography, is thought to achieve better imaging of the interstitial part of fallopian tubes and significantly contribute to the diagnosis of interstitial ectopic pregnancy [16]. Furthermore, three-dimensional magnetic resonance imaging is considered to be of utmost help in differentiating interstitial ectopic pregnancy from angular pregnancy. In these cases, early diagnosis and avoidance of unnecessary interventions are crucial, contributing significantly to the prevention of hemorrhage and the reduction of morbidity and mortality [17]. Therefore, based on clinical findings, imaging results, and quantification of beta-chorionic gonadotropin hormone levels, the diagnosis of interstitial ectopic pregnancy can be established earlier and more accurately, providing the advantage of prompt treatment [18].

The choice between pharmaceutical and surgical treatment for interstitial ectopic pregnancy depends on factors such as the timing of the initial diagnosis, the overall condition of the patient, her desire to preserve fertility, the availability of medical equipment, and the surgical skills of the medical team [19]. Methotrexate and mifepristone are the most common medications occasionally used to treat interstitial ectopic pregnancy. Methotrexate, administered either topically or systemically, in single-dose or multi-dose regimens, is the most widely used drug and has shown the best therapeutic outcome [20]. The combined administration of methotrexate and mifepristone is considered a safe therapeutic option, especially for patients with elevated levels of the beta-chorionic gonadotropin hormone. However, treatment should be individualized, taking into account factors such as the patient's gestational age, clinical presentation, and her desire for a future pregnancy. For the treatment of uterine arteriovenous malformation, which may occur after medication administration, uterine artery embolization is recommended and has shown favorable treatment outcomes [9, 21]. In our case, the lack of experience in the conservative management of the disease, involving ultrasound-guided local injection of methotrexate or systemic administration of the drug, combined with the patient's lack of desire to preserve fertility and achieve future pregnancy, led to the decision for surgical treatment through laparotomy. The laparoscopic approach was not available at our hospital.

The standard surgical management of interstitial ectopic pregnancy includes wedge resection of the affected uterine horn with simultaneous ipsilateral salpingectomy and exploratory laparotomy, which are associated with a high risk of massive hemorrhage and an increased possibility of performing a hysterectomy [22]. However, remarkable improvements in ultrasound imaging techniques achieved in recent years now allow early diagnosis of interstitial ectopic pregnancy and, simultaneously, provide the potential for a more conservative surgical treatment of the disease. In those cases where the woman's future plans include the desire to achieve pregnancy and the clinical condition allows a laparoscopic approach to the disease, laparoscopic cornuostomy seems to be the best treatment option [23]. Currently, it is estimated that a laparoscopic approach to interstitial ectopic pregnancy can be safely recommended, even in patients with advanced gestational age [24]. Additionally, conservative methods such as hysteroscopic resection of the ectopic pregnancy and laparoscopically-guided aspiration of the gestational sac through the cervix have been reported in the international literature to be successfully applied, although in a very small number of cases. The method is less traumatic and has the advantage of preserving the fallopian tubes and maintaining the integrity of the myometrium, allowing the woman to resume attempts at conception sooner [25, 26]. Finally, even currently, hysterectomy is still the last option in the treatment of interstitial ectopic pregnancy, especially when it concerns an advanced pregnancy with extensive rupture of the uterine wall accompanied by major, life-threatening hemorrhage [27].

## Conclusions

Interstitial ectopic pregnancy with painless, severe uterine bleeding is extremely rare. The scarcity of cases and the challenges in preoperative diagnosis contribute to the absence of established guidelines for the management of these patients. Therefore, early recognition of both classical and non-specific symptoms related to the disease, coupled with the correct use of modern diagnostic technology, can play a crucial role in achieving early diagnosis and facilitating the immediate application of optimal, up-to-date treatment options. It is considered that the early and accurate preoperative diagnosis of interstitial ectopic pregnancy represents a critical step in the successful management of this rare but potentially life-threatening obstetric complication.
